# The Ratio and Concentration of Two Monoterpenes Mediate Fecundity of the Pinewood Nematode and Growth of Its Associated Fungi

**DOI:** 10.1371/journal.pone.0031716

**Published:** 2012-02-20

**Authors:** Hongtao Niu, Lilin Zhao, Min Lu, Shuai Zhang, Jianghua Sun

**Affiliations:** 1 State Key Laboratory of Integrated Management of Pest Insects and Rodents, Institute of Zoology, Chinese Academy of Sciences, Beijing, China; 2 Graduate University of Chinese Academy of Sciences, Beijing, China; University of Wisconsin-Milwaukee, United States of America

## Abstract

The pinewood nematode (PWN) *Bursaphelenchus xylophilus*, vectored primarily by the sawyer beetle, *Monochamus alternatus*, is an important invasive pest and causal agent of pine wilt disease of Chinese Masson pine, *Pinus massoniana*. Previous work demonstrated that the ratios and concentrations of α-pinene∶β-pinene differed between healthy trees and those trees containing blue-stain fungus (and *M. alternatus* pupae). However, the potential influence of the altered monoterpene ratios and concentrations on PWN and associated fungi remained unknown. Our current results show that low concentrations of the monoterpenes within petri dishes reduced PWN propagation, whereas the highest concentration of the monoterpenes increased PWN propagation. The propagation rate of PWN treated with the monoterpene ratio representative of blue-stain infected pine (α-pinene∶β-pinene = 1∶0.8, 137.6 mg/ml) was significantly higher than that (α-pinene∶β-pinene = 1∶0.1, 137.6 mg/ml) representative of healthy pines or those damaged by *M. alternatus* feeding, but without blue stain. Furthermore, inhibition of mycelial growth of associated fungi increased with the concentration of the monoterpenes α-pinene and β-pinene. Additionally, higher levels of β-pinene (α-pinene∶β-pinene = 1∶0.8) resulted in greater inhibition of the growth of the associated fungi *Sporothrix* sp.2 and *Ophiostoma ips* strains, but had no significant effects on the growth of *Sporothrix* sp.1, which is the best food resource for PWN. These results suggest that host monoterpenes generally reduce the reproduction of PWN. However, PWN utilizes high monoterpene concentrations and native blue-stain fungus *Sporothrix* sp.1 to improve its own propagation and overcome host resistance, which may provide clues to understanding the ecological mechanisms of PWN's successful invasion.

## Introduction

Monoterpenes (a class of C_10_ terpenes that consist of two isoprene units) are volatile compounds that occur in conifers and other plants. Many monoterpenes can increase exponentially in response to fungal pathogen and insect herbivore attack [1]–[5]. A number of inducible terpenoids play important roles in mediating interactions between plants and other organisms [6]–[8]. For example, monoterpenes have complex functions in conifer-bark beetle-fungal interactions, which may thereby enhance the invasion success of red turpentine beetle (*Dendroctonus valens* LeConte) by the beetle-fungal complex [9]. Both α-pinene and volatiles from some bacterial associates are capable of stimulating the growth of the native American fungus *Leptographum procerum*, resulting in complex interactions between host volatiles, bacteria, and fungi that can affect *D. valens* and *D. ponderosae* fitness [10]. The monoterpene present in induced reaction tissue can be toxic to adult beetles (e.g. *Ips pini*) and their brood, and inhibitory to their associated fungi [2], [11]. Furthermore, monoterpenes function in plant defense. For example, monoterpenes synthesized by plants have antifungal and antibacterial properties [2], [12]–[14] and are toxic to other organisms [7], [15], [16].

The pinewood nematode, (PWN), *Bursaphelenchus xylophilus* (Steiner et Buhrer) Nickle, is the causal agent of pine wilt disease [17]–[18]. PWN, an invasive species native to North America, is primarily vectored by the sawyer beetle, *Monochamus alternatus* Hope (Coleoptera: Cerambycidae). In its introduced range in China and Japan, PWN is highly invasive and disruptive to Masson pine (*Pinus massoniana* Lamb.), which is one of the most widely planted conifers in Asia [18]–[20]. Maturation feeding and the pupal stage of *M. alternatus* are two important vector phases in the development of pine wilt disease, which is associated with PWN's phytophagous and mycophagous stages. PWN is initially phytophagous, entering healthy host trees via wounds caused during maturation feeding of *M. alternatus* and feeding on the epithelial cells of the resin ducts [18], [21]–[22]. During its mycophagous stage, PWN feeds upon fungi inhabiting the dead trees, then accumulate around *M. alternatus* pupal chambers, migrate onto the vector and are subsequently carried by emergent *M. alternatus* adults to new host trees [23]–[24]. The dying or dead tree is colonized by various wood-inhabiting fungi, including blue-stain fungi, which provide a further food source for PWN [25]–[26]. Blue-stain fungi cause blue-staining in trees and greatly affect the number of PWN carried by the vector beetles [27]–[28]. Extensive evidence has demonstrated the nematicidal activity of monoterpenoids against PWN [29]–[31]. Moreover, the specific ratio of monoterpenes has been shown to influence the PWN behavior [32]–[33]. Besides these effects on nematodes, monoterpenes have also been shown to inhibit the mycelial growth of blue-stain fungi [13], [34]–[36].

The monoterpenes α-pinene and β-pinene represent the main constituents of Masson pine, and their relative amounts and proportions change in relation to the progression of pine wilt disease [32], [37]–[38]. Previous studies of monoterpenes in Masson pine have revealed that ratios and concentrations of α-pinene∶β-pinene differed between healthy trees and those containing blue-stain fungus (and *M. alternatus* pupae) [32]. The extent to which the altered concentrations and ratios of monoterpenes influence PWN propagation and associated fungal growth during the two important phases of its vector beetle remains unknown. To investigate this question, we assessed the change of the monoterpenes α-pinene and β-pinene in Masson pine caused by feeding by *M. alternatus* adults. Then we simulated the specific monoterpene ratios and concentrations representative of healthy pines, blue-stain infected pupation chambers of *M. alternatus*, and pines fed on by *M. alternatus* adults, and examined their effects on propagation of PWN and the mycelial growth of three strains of blue-stain fungal associates. We also tested PWN propagation on these three strains of blue-stain fungi.

## Results

### Volatiles analysis

Analysis of the changes in the monoterpenes of Masson pine by GC revealed that *M. alternatus* feeding significantly increased concentration of α-pinene in pine branch by 1.21 times (*F*_1, 8_ = 30.779; *p* = 0.041), it has little influence on β-pinene concentrations (*F*_1, 8_ = 0.075, *p* = 0.623) (Table 1).

**Table 1:** Changes in the monoterpenes of Masson pine material after *Monochamus Alternatus* feeding for 24 h.

Treatment	Concentration (mg/ml)	
	α-pinene (means ± SE)	β-pinene (means ± SE)
**Pine material before feeding**	0.0813±0.00261b	0.0120±0.00661a
**Pine material after feeding**	0.170±0.0332a	0.0162±0.00598a

Means in a column followed by different letter are significantly different at *p*<0.05, data were analyzed by a *t*-test for independent samples.

### Concentration and ratio of α-pinene and β-pinene on the propagation of PWN

**Figure 1:**
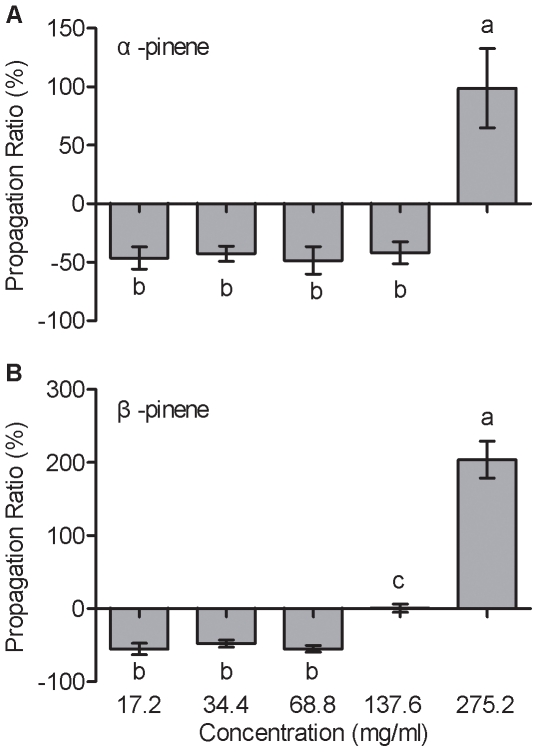
Propagation responses to different concentrations of α-pinene and β-pinene by pinewood nematode (PWN) using the cotton ball bioassay. (A) α-pinene (17.2, 34.4, 68.8, 137.6, and 275.2 mg/ml), (B) β-pinene (17.2, 34.4, 68.8, 137.6, and 275.2 mg/ml). Each data bar represents the mean of eight independent replicates, and error bars represent standard errors of the mean. Different lowercase letters above or below bars indicate significant differences (Tukey's multiple comparison test; *p*<0.05).

Propagation responses of PWN varied according to the concentrations of both α-pinene and β-pinene (Figure 1). Results indicate that there is no significant difference between low concentrations from 17.2 to 137.6 mg/ml (*p*>0.05), except for the concentration of β-pinene, 137.6 mg/ml (*p*<0.05; Figure 1B). At the highest concentration of 275.2 mg/ml, the propagation of PWN was significantly stimulated (α-pinene, Propagation Ratio (PR) = 98.80%; β-pinene, PR = 204.14%), and treatment with β-pinene had significantly higher propagation ratios than treatments with α-pinene (*F*_1, 14_ = 0.815, *p* = 0.026). PWN propagation ratios were significantly higher at the highest monoterpene concentration compared to all other concentrations (Figure 1A, *F*_4, 39_ = 13.686, *p*<0.05; Figure 1B, *F*_4, 39_ = 80.763, *p*<0.05). In addition, the propagation rate of PWN treated with the resin ratio representative of blue-stain infected pine (α-pinene∶β-pinene = 1∶0.8, 137.6 mg/ml) was significantly higher (15.10%) than those treated with resin ratio associated with healthy pine/pine damaged by *M. alternatus* feeding (α-pinene∶β-pinene = 1∶0.1, 137.6 mg/ml) (*F*_1, 14_ = 8.953, *p* = 0.01).

### Concentration and ratio of α-pinene and β-pinene on mycelial growth

**Figure 2:**
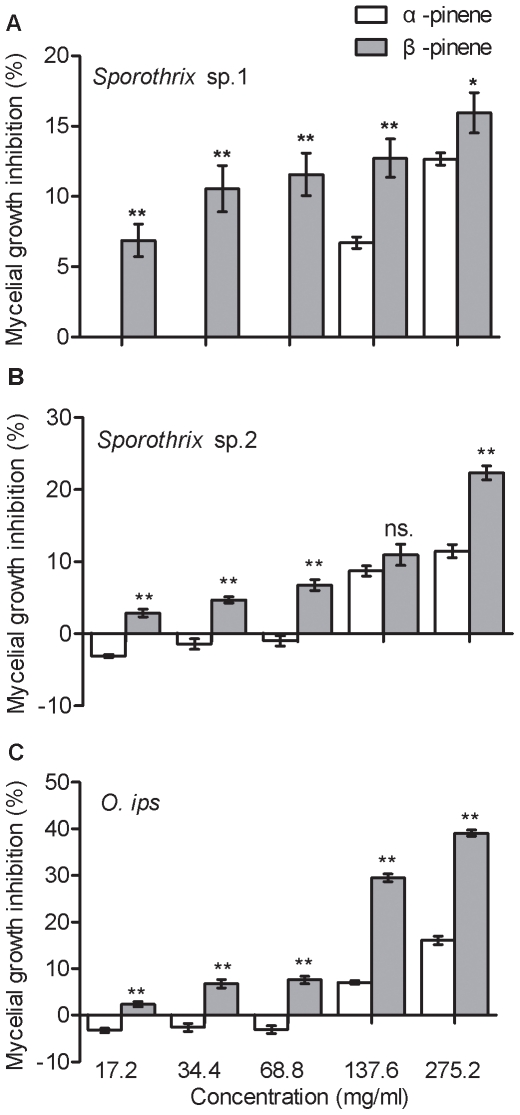
Differences between the mycelial growth inhibition of blue-stain fungal strains (A *Sporothrix* sp.1, B *Sporothrix* sp.2, and C *O. ips*) on 2% malt extract agar in the presence of α-pinene and β-pinene at the same concentration. Data were analyzed by a *t*-test for independent samples, for each blue-stain fungal strain in the presence of α-pinene and β-pinene at the same concentration. Each data bar represents the mean of eight independent replicates, and error bars represent standard errors of the mean (** *p*<0.01, * *p*<0.05 and ns. *p*>0.05).

Results indicated that α-pinene and β-pinene inhibited mycelial growth of *Sporothrix* sp.1, *Sporothrix* sp.2, and *Ophiostoma ips*, and the effect was concentration-dependent (Figure 2). All concentrations of α-pinene alone showed weak activity, and there was a strong and significant correlation between the mycelial growth inhibition of fungal strains and the concentrations of α-pinene (y = 0.0532x-1.7975, r^2^ = 0.9561, *p* = 0.004 for *Sporothrix* sp.1; y = 0.0595x-3.4142, r^2^ = 0.8810, *p* = 0.018 for *Sporothrix* sp.2; y = 0.0800x-5.6840, r^2^ = 0.9571, *p* = 0.004 for *O. ips*). At the same concentrations, β-pinene exhibited stronger antifungal activities than α-pinene for each fungus (Figure 2) and the correlations between the inhibition of mycelial growth of fungal strains and the concentrations of β-pinene were also significant (y = 0.0288x+8.4650, r^2^ = 0.8376, *p* = 0.029 for *Sporothrix* sp.1; y = 0.0739x+1.6408, r^2^ = 0.9954, *p*<0.001 for *Sporothrix* sp.2; y = 0.148x+1.2992, r^2^ = 0.9170, *p* = 0.01 for *O. ips*).

**Figure 3:**
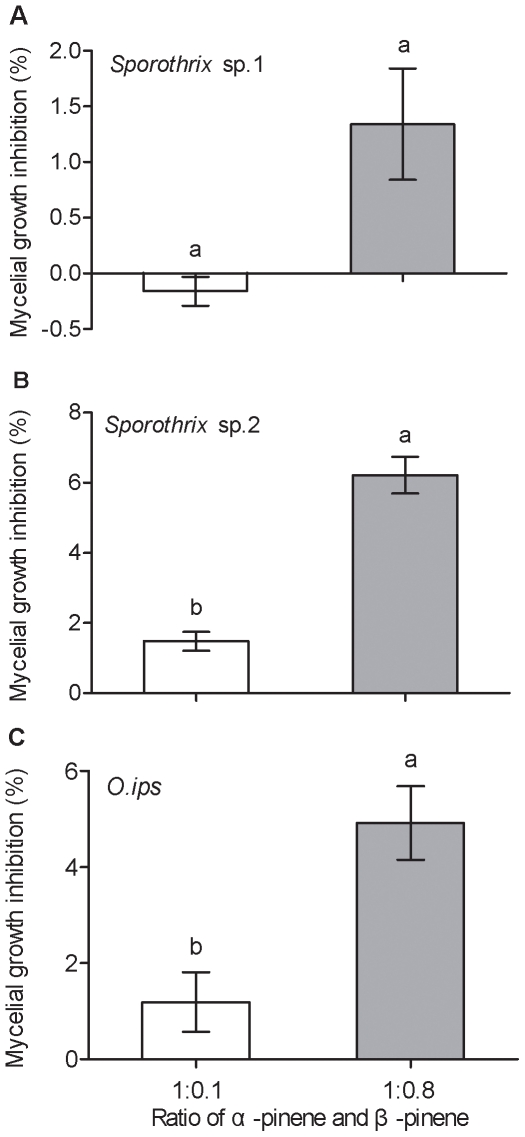
Inhibition of mycelial growth of blue-stain fungus on 2% malt extract agar in the presence of different ratios of α-pinene and β-pinene at 137.6 mg/ml concentration. (A) *Sporothrix* sp.1, (B) *Sporothrix* sp.2 and (C) *O. ips*. Data were analyzed by a *t*-test for independent samples for each blue-stain fungal strain. Significant differences within species are indicated by different lowercase letters (*p*<0.05). Values represent the means and standard errors of eight replicates for each treatment.

The resin ratio of α-pinene to β-pinene representative of blue-stain infected pine had a more significant negative effect than resin ratios associated with healthy pine/pine damaged by *M. alternatus* feeding on mycelial growth (Figure 3). Although the mycelial growth inhibition of *Sporothrix* sp.1 was less effective (*F*_1, 14_ = 1.944, *p* = 0.315; Figure 3A), there were significant differences in the mycelial growth inhibition of *Sporothrix* sp.2 (*F*_1, 14_ = 0.233, *p* = 0.009; Figure 3B) and *O. ips* (*F*_1, 14_ = 0.438, *p*<0.001; Figure 3C) between the resin ratio of α-pinene∶β-pinene, 1∶0.1 and the resin ratio of α-pinene∶β-pinene, 1∶0.8 treatments, respectively.

### Propagation of nematodes on three blue-stain fungal strains

**Figure 4:**
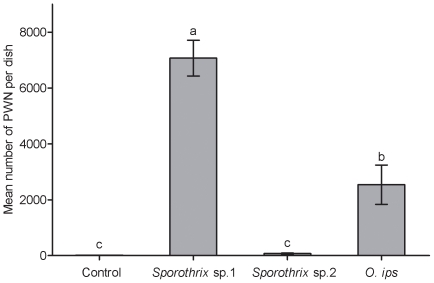
Population growth of pinewood nematode on three blue-stain fungi. Nematode numbers were counted ten days after inoculation in the fungal culture at an initial number of forty individuals per dish. Values represent the means and standard errors of eight replicates for each treatment. Different lowercase letters above bars indicate significant differences (Tukey's multiple comparison test, *p*<0.05).

There were significant differences in the growth of PWN on *Sporothrix* sp.1, *Sporothrix* sp.2 and *O. ips* strains (*F*_3, 28_ = 48.488, *p*<0.05; Figure 4). The mean number of nematodes was significantly higher on *O. ips* (mean ± SE, 2539.4±703.3) than on *Sporothrix* sp.2 (mean ± SE, 74.1±20.8) and controls (mean ± SE, 14.5±2.7), which was significantly less than that on *Sporothrix* sp.1 (mean ± SE, 7074.4±641.4, *p*<0.001). No significant statistical differences were found in the total numbers of nematodes between *Sporothrix* sp.2 and controls (*p* = 0.930).

## Discussion

Chemical analysis of Masson pine volatiles from pine material before and after *M. alternatus* feeding revealed a significantly higher concentration of α-pinene in the pine material after feeding compared to control twigs, but there were no significant differences in β-pinene concentrations (Table 1). This finding is similar to that reported in other studies of insect damage Masson pine [38]. The accumulation of high levels of monoterpenes by conifers in response to herbivore and pathogen attack is an important component of the host defense system, which is associated with terpene synthesis [7], [12]. In contrast, for the *M. alternatus* pupal phase, β-pinene levels in blue-stained pine samples were significantly higher than those of healthy samples, while α-pinene was lower in blue-stained pines than in healthy trees [32]. Our results suggest that the changes of the ratios and concentrations of with respect to these two vector phases influence the progression of pine wilt disease through the relations of the PWN and associated fungi, and may provide clues to understanding the invasion mechanism.

The growth of many species of nematodes such as *Caenorhabditis elegans* [39], *Haemonchus contortus* [40], and the PWN [30], can be inhibited by monoterpenes. Our experiments indicated that PWN propagation ratios were significantly increased by the highest monoterpene concentration treatment (Figure 1). In general, healthy *P. taeda* stands growing in North America are highly resistant to PWN and contain a higher concentration of volatile terpenoids than *P. thunbergii* [41]. Whereas the susceptible species (*P. densiflora*) was completely destroyed, highly resistant species (*P. taeda*) can be damaged by PWN [23]. So PWN may have ability to utilize high concentrations of volatile terpenoids to overcome hosts. During pupation of and maturation feeding by *M. alternatus* adults in Masson pine, both wood with blue-stain fungi and branches with feeding wounds exhibited increased monoterpene concentrations. This suggests the possibility that PWN may retain their niches and related ecological traits over time, to make use of elevated concentrations of terpenoids, and this is consistent with previous findings that demonstrated that the niche requirements in its native range matching habitat availability in the new environment should enhance invasion success [42]–[43]. In addition, the fact that the propagation rate of PWN treated with the resin ratio of α-pinene and β-pinene (1∶0.8) representative of blue-stain infected pine was significantly higher (15.10%) than those treated with resin (1∶0.1) associated with healthy pine/pine damaged by *M. alternatus* feeding suggests that tree defenses are likely to impose strong natural selection on nematode populations, indicating that individuals adapted to the altered ratio will have more offspring. Therefore, the altered concentrations and ratios of the monoterpenes α-pinene and β-pinene may directly increase PWN populations, which play an important in helping the PWN overcome its hosts' defenses and successful invasion [44]. Further experiments are needed to test the effects of the monoterpenes released from both *P. taeda* (a native host) and *P. massoniana* (a non-native host) on PWN to fully understand the propagation mechanisms of PWN.

We demonstrated that changes in the monoterpenes of Masson pine inhibit the mycelial growth of fungal strains at the concentrations observed during these two important vector phases of *M. alternatus*, which is similar to previous research [13], [34]–[36]. The strains *Sporothrix* sp.1, *Sporothrix* sp.2, and *O. ips* are also suitable for PWN propagation (Figure 4), which may indirectly reduce PWN potential colonization. The inhibition of fungal growth is positively correlated to concentrations of the monoterpenes α-pinene and β-pinene (Figure 2). Moreover, our results show that each fungus may respond differently to changing concentrations of each monoterpene, which is consistent with previous results [45]. At the same concentrations, β-pinene exhibited stronger antifungal activities than α-pinene for each fungus (Figure 2). However, β-pinene increases in the blue-stain infected samples associated with the pupal stage of *M. alternatus* [32], which may indirectly reduce the propagation of PWN since blue-stain fungi are one of main food sources for PWN. In general, volatiles emitted by plants are always a mixture of several compounds released in response to the invading pathogens [7]–[8]. However, the altered ratios of α-pinene and β-pinene did not inhibit the mycelial growth of the blue-stain fungus *Sporothrix* sp.1, but did inhibit growth of *Sporothrix* sp.2 and *O. ips* (Figure 3). The blue-stain fungus *Sporothrix* sp.1 grew fastest and generated the highest population growth of PWN, which provides a huge number of PWN to be vectored by *M. alternatus* to new host trees.

Why was the PWN so successful in its invasion of China and Japan? Previous studies have shown that a number of factors contribute to invasion success of exotic species, and several hypotheses have been proposed, including phytotoxins, cellulose, PWN and bacteria, and terpenoids [46]–[49]. PWN is an excellent model organism to study the complex native multitrophic species interactions in its introduced range, including susceptible native tree species (Masson pine), a native insect vector (*M. alternatus*), and native associated fungi. PWN has become a highly destructive and invasive forest pest. Our study focused on the potential influence of the altered monoterpenes during the two important vector phases on PWN and associated fungi. The results indicate that high concentrations of monoterpenes could increase the propagation of PWN and decrease growth of its associated fungi, which may provide clues to understanding the ecological mechanisms of PWN's successful invasion. The monoterpene concentrations we used were higher than those we found to occur in trees. As such, the exact role of naturally occurring monoterpenes in the PWN system remains worthy of further exploration.

## Materials and Methods

### Source of *Monochamus alternatus*, nematodes and fungi

*M. alternatus* were harvested in March 2010 from naturally infested, dead Masson pine trees in Zhejiang Province. Bolts were placed in outdoor cages and emerging adults were collected daily in June 2010. *M. alternatus* pupal chambers, also collected from dead Masson pine trees in Zhejiang province in 2007, were placed in Baermann funnels to recover the nematodes. The nematodes were washed 3 times in M9 buffer (42.3 mM Na_2_HPO_4_, 22 mM KH_2_PO_4_, 85.6 mM NaCl, and 1 mM MgSO_4_, pH 7.0) [50], 3 times in sterile water, and were then cultured with the fungus *Botrytis cinerea* grown on 2% malt extract agar (MEA, 7 g Biolab malt extract, 7 g Biolab agar, and 350 ml deionised water, pH = 5.75). Prior to experiments, nematodes were rinsed from the culture dish lids with distilled water. Three fungal strains, *Sporothrix* sp.1 (CMW29982), *Sporothrix* sp.2 (CMW29978), and *O. ips* (CMW29981) were identified from pupal chambers of *M. alternatus* from *Bursaphelenchus xylophilus*–infested Masson pine in Zhejiang in 2007, China (our unpublished data). *Sporothrix* sp.1 and *Sporothrix* sp.2 are new, undescribed species in China. All cultures used in this study are maintained in the culture collection (CMW) of the Forestry and Agricultural Biotechnology Institute (FABI), University of Pretoria, Pretoria, South Africa.

### *Monochamus alternatus* feeding and monoterpene analysis

Newly emerged *M. alternatus* were isolated, starved for 12 h, then reared by sleeving them with stainless steel mesh on the twigs of living Masson pine trees (five year old trees with height 180–200 cm and stem diameters 2.5–3.0 cm). Controls consisted of healthy Masson pine trees which did not have feeding by *M. alternatus*. After 24 h, approximately 500 mg of pine twigs with feeding wounds and healthy pine twigs were cut into ∼2 cm sections, and the entire branch sections were extracted with 4 ml hexane for 12 hr. Following extraction, the extracts were filtered through glass wool, and stored at −20°C. Just prior to GC (Agilent 7890A) analysis, extracts were filtered through 0.45 µm Teflon syringe filters.

The GC was fitted with a DB-WAX column (60 m length×0.25 mm i.d.×0.25 µm film, J&W Scientific, Folsom, CA, USA) and flame ionization detector (FID). The on-column injector temperature was 220°C and nitrogen (99.999%) was used as carrier gas (flow rate, 1 ml/min). The column temperature was programmed from an initial temperature of 50°C for 1 min, increased to 80°C at 3°C/min, increased to 120°C at 5°C/min, and then ramped to 230°C at 10°C/min (10 min hold time). Splitless injections of 1 µl were used for analyses, and mixtures of α-pinene and β-pinene were used as external standards. The identification of the chemical components of each sample was based on a comparison of their retention times with these standards. The concentrations of α-pinene and β-pinene were determined by extrapolation to external standard calibration curves of α-pinene and β-pinene (0.01, 0.025, 0.05, 0.25 and 0.5 mg/ml; r^2^ = 0.9999 for α-pinene and r^2^ = 0.9997 for β-pinene), respectively. All quantifications were calculated on calibrated GC-FID peak areas.

### Monoterpene preparation

The monoterpenes α-pinene (Acros Organics, Purity 97%) and β-pinene (Alfa Aesar, Purity 99%) were chosen for this study because they represent the primary terpenoid constituents of Masson pine [32], and each was tested at five concentrations (17.2, 34.4, 68.8, 137.6, and 275.2 mg/ml) in hexane (Fisher chemicals, Purity 99%). The ratios of α-pinene and β-pinene used in experiments were 1∶0.1 (representative of healthy pine/pine damaged by *M. alternatus* feeding) and 1∶0.8 (representative of blue-stain infected pine) with the concentration of 137.6 mg/ml. These concentrations were chosen because both previous studies and our current study determined that these concentrations of α-pinene and β-pinene are representative of healthy pine samples, pine material after feeding, and blue-stain samples of *P. massoniana*, and thus provided a gradient of monoterpene concentration from which to test the propagative nematode's behavioral response [32]–[33], [37].

### Concentration and ratio of α-pinene and β-pinene on propagation of PWN

A cotton ball bioassay was used to determine whether α-pinene and β-pinene exhibited propagation inhibition or stimulation toward PWN [30], [51]. Monoterpenes were prepared as described above, and the fungus *B. cinerea* was grown on 2% MEA. When the fungal colonies covered the petri dish (35 mm diameter), a mycelial disc (5 mm diameter) was taken out from the MEA medium center and placed in a suitable-sized sterile cotton ball (diameter approximately 5 mm). At this point, a 40 µl aqueous nematode suspension of eighty nematodes (juveniles∶adults = 40∶40) was injected. Subsequently, 50 µl of monoterpene solution were injected into the sterile cotton balls (eight replicates of each). Controls were injected with 50 µl of hexane. The dishes were then sealed with parafilm (Pechiney Plastic Packaging, Menasha, WI, USA) and incubated in the dark at 25°C for ten days. The living nematodes were separated from the culture by the Baermann funnel technique and counted [52].

### Concentration and ratio of α-pinene and β-pinene on mycelial growth

To determine the effects of α-pinene and β-pinene on the growth of fungi, three fungal strains, i.e., *Sporothrix* sp.1, *Sporothrix* sp.2, and *O. ips*, were tested with a modified method of Hofstetter et al. (2005) [36]. A mycelial disc (5 mm diameter) was taken out from the periphery of an actively growing culture on 2% MEA for ten days and placed at the center of a 90×20 mm petri dish containing 20 ml of 2% MEA. For each treatment, 1 ml of test solution was added to a sterile filter paper (80 mm diameter) and the paper was placed onto the lid of a petri dish. Plates were rapidly sealed with parafilm and incubated at 25°C in darkness. Eight petri dish cultures for each treatment were used, including two controls (filter paper with 1 ml hexane and filter paper only). Radial growth was measured in four directions (0°, 90°, 180°, 270°) for each plate with the interval of 24 h for *Sporothrix* sp.1 and 48 hr for the other two until fungus reached the end of the petri dish. Tests were continued until one treatment of the fungus reached the end of the petri dish. Mean values from the final measurement of each dish were used for statistical analysis. Growth inhibition of treatment against control was calculated as a percentage, using the following formula: 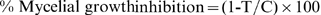, where C is hyphal extension (mm, radius) of solvent controls and T is hyphal extension (mm, radius) of plates treated with monoterpenes [53].

### Propagation of nematodes on three blue-stain fungal strains

Population growth tests of nematodes were conducted with three blue-stain fungal strains (*Sporothrix* sp.1, *Sporothrix* sp.2, *O. ips*) grown on 2% MEA (2% MEA medium without fungus as control). When the fungal colonies covered the whole petri dish (35 mm diameter), eighty nematodes (juveniles∶adults = 40∶40) suspended in 40 µl of sterile water were inoculated in the middle of each dish (8 replicates). 10 days after the dishes were incubated at 25°C in darkness, nematodes were destructively sampled and separately extracted by the Baermann funnel technique [52].

### Statistical Analyses

Data were analyzed with the statistical software SPSS 17.0 for Windows (SPSS Inc., Chicago, IL, USA). We used a *t*-test for independent samples to compare changes in Masson pine monoterpenes between infested (*M. alternatus*) pines and healthy pines, and also compared the mycelial growth inhibition of each blue-stain fungus treated with different ratios and concentrations of α-pinene and β-pinene. The relationship between the relative mycelial growth rate of each blue-stain fungus and the concentration of α-pinene or β-pinene was determined by linear regression [54]. The Propagation Ratio (PR) was determined using the formula: 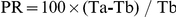 where T_a_ is the number of nematodes after chemical treatment and T_b_ is the number of nematodes after hexane treatment. Propagation ratio, mycelial growth inhibition and nematode population growth were analyzed with ANOVA and means were separated with Tukey's multiple comparison test.

### Ethics statement

All necessary permits were obtained for the described field studies from the Forest Pest Control and Quarantine Bureau of Zhejiang Province. These field studies do not involve endangered or protected species.
